# In2AB Janus Double-Layered Honeycomb Structures with Nontrivial Topology and Rashba Splitting (A, B = P, As, Sb, or Bi)

**DOI:** 10.1021/acsomega.5c07907

**Published:** 2025-11-10

**Authors:** Joel D’Souza, Ina Marie R. Verzola, Rovi Angelo B. Villaos, Aniceto B. Maghirang, Zhi-Quan Huang, Yijun Shi, Feng-Chuan Chuang

**Affiliations:** † Department of Physics, 34874 National Sun Yat-sen University, Kaohsiung 80424, Taiwan; ‡ Division of Machine Elements, Luleå University of Technology, Luleå SE 97187, Sweden; § Applied Physics, Division of Materials Science, Department of Engineering Sciences and Mathematics, Luleå University of Technology, Luleå SE 97187, Sweden; ∥ Physics Division, National Center for Theoretical Sciences, Taipei 10617, Taiwan; ⊥ Center for Theoretical and Computational Physics, National Sun Yat-sen University, Kaohsiung 80424, Taiwan; # Department of Physics, National Tsing Hua University, Hsinchu 30013, Taiwan

## Abstract

Two-dimensional (2D) systems serve as promising platforms for engineering nanoscale devices and for exploring emergent quantum phenomena. The recent experimental realization of AlSb in a double-layer honeycomb (DLHC) configuration confirms the prior theoretical predictions that traditional semiconductors are stable in DLHC configurations at the ultrathin limit and paves the way for systematic exploration of structurally robust DLHC materials. In this work, we have studied In-based InA (A = P, As, Sb, or Bi) in wurtzite, zincblende, DLHC, and AA configurations and found that all the pristine InA structures energetically prefer the DLHC structure. The Z_2_ topological invariant number calculation showed that the pristine case is topologically trivial. Furthermore, using the energetically favored configuration, we tailored a Janus In_2_AB (A and B = P, As, Sb, or Bi) that breaks the inversion symmetry. Topological property calculations under the hybrid HSE06 functional reveal that three out of six compounds (In_2_PSb, In_2_AsSb, and In_2_BiSb) exhibit nontrivial topology with system band gaps of 527, 456, and 649 meV, respectively. The presence of gapless edge states further confirmed the nontrivial topological property of these compounds. Moreover, In_2_PSb also showed a significant isotropic Rashba splitting, with a Rashba parameter of α_R_ = 1.45 eV Å. These materials with nontrivial topology and Rashba-like splitting might have meaningful applications in spintronics.

## Graphical abstract

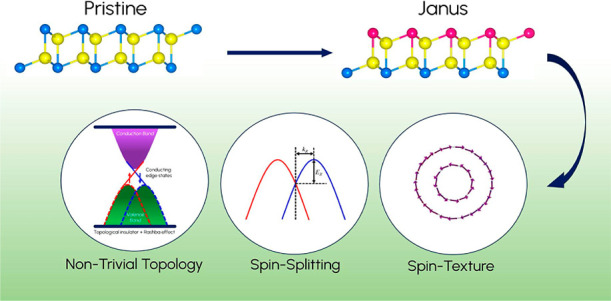

## Introduction

Two-dimensional (2D) materials have earned a lot of attention because of their distinctive physical features, such as spin-valley coupling,^1^ quantum spin Hall state,^2^ and Ising ferromagnetism,^3^ presenting effective alternatives for use in electronic devices of the next generation. The study of 2D materials, driven by the breakthrough discovery of graphene,^4^ holds the key to unraveling the quantum phenomenon that could find applications in multifunctional quantum devices. This significant milestone eventually paved the way for Kane and Mele’s model, which showed the nontrivial topological^5^ behavior due to the spin–orbit coupling (SOC) interaction. The experimentally synthesized graphene inspired the research of materials having a honeycomb structure that demonstrated nontrivial topology.^6–12^ Additionally, it expanded the field of 2D materials to magnetic^13–16^ and nonmagnetic systems by showing properties that are different from those of their three-dimensional^17,18^ (3D) counterparts.

The advancements in theoretical simulations and synthesis techniques have made it possible to investigate 2D materials beyond graphene.^19^ This includes materials like MXenes,^20^ black phosphorus,^21^ Zintl^22^ compounds, and transition metal dichalcogenides.^23–26^ Notably, the majority of these materials are van der Waals (vdW) 2D materials, which can be directly exfoliated from their bulk counterparts.^27^

Among the plethora of 2D materials, an intriguing class of materials known as 2D topological insulators (2D TIs) features an insulating bulk along with symmetry-protected edge states. These edge states are restricted to a single conducting channel while exhibiting minimal backscattering, resulting in a considerable reduction of heat dissipation.^28,29^ These features make 2D TIs promising candidates for applications in low-power electronics, spintronics, and quantum computing.^29,30^ Driven by these unique properties, numerous 2D materials have been predicted to exhibit topological insulating behavior.^22,31–35^ As interest in this field continues to grow, the demand is increasing for materials that are not only topological insulators but also easy to synthesize and capable of operating at room temperature. Meanwhile, after the successful synthesis of 2D MoSSe, which was achieved through the selenization of MoS_2_ ^36^ and sulfurization of MoSe_2_,^37^ there was a shift in research focus from pristine to Janus 2D materials. Broken inversion symmetry is the main characteristic of a Janus material, which can be accomplished via functionalization,^37,38^ atomic substitution,^25,39^ or heterostructures,^40,41^ which enables the emergence of the Rashba effect. This effect introduces a momentum-dependent spin-splitting in the electronic structure of 2D materials that possess structural asymmetry.^42^

Moving on, a particular class of 2D materials known as double-layered honeycomb (DLHC), where two honeycomb layers like graphene are bonded in a way that stabilizes the material, was theoretically demonstrated to be another possible stable structure for most of the traditional semiconductors (I–VII, II–VI, and III–V semiconductors) in the ultrathin limit.^43^ This was followed by the synthesis of 2D AlSb in the DLHC configuration, strengthening the viability of the theoretically predicted DLHC materials. Moreover, 2D CuI and AgI were stabilized in the DLHC configuration in normal conditions by growing them on graphene encapsulation.^44^ Furthermore, these 2D DLHC materials are potentially known to host physical properties, such as the excitonic insulator phase and topological properties.^45^

In this work, we explored the energetically preferred In-based DLHC structure and tailored a Janus In_2_AB DLHC structure obtained by replacing the top layer of A atoms with B atoms. Specifically, we investigated the electronic, topological, and spin properties and established their structural stability. Our results demonstrated the possibility of tuning the nontrivial topology and Rashba-like splitting properties of 2D DLHC compounds by breaking their inversion symmetry via Janus formation, which might have meaningful applications in spintronics.

## Methods

In this study, first-principles calculation in the framework of density functional theory (DFT)^46^ as implemented in the Vienna Ab initio Simulation Package (VASP)^47,48^ was performed to investigate the electronic and topological properties, as well as the structural stability. The exchange–correlation effects were treated using generalized gradient approximation in Perdew–Burke–Ernzerhof (GGA-PBE)^49–51^ and projector-augmented wave (PAW)^52,53^ with an energy cutoff of 400 eV. A 15 Å vacuum was added along the *z*-direction. The structure was optimized until the residual force on each atom was less than 10^–4^ eV/Å, with a convergence criterion of 1 × 10^–6^ eV/Å. The Brillouin zone was sampled using a Γ-centered Monkhorst–Pack^54^ grid of 18 × 18 × 1. The HSE06 functional^55,56^ was used to predict the bandgap of the material accurately. Therefore, in addition to GGA-PBE, the electronic band structure, topological invariance (Z_2_), and edge states were computed using the HSE06 functional. The dynamic stability was confirmed by calculating the phonon spectra using the PHONOPY^57^ package with a large 4 × 4 × 1 supercell. Furthermore, to confirm the thermal stability, ab initio molecular dynamics (AIMD) simulations were carried out based on the Nosé thermostat,^58^ with a supercell size of 4 × 4 × 1. The AIMD simulations were performed for 10 ps with a 1 fs time step at 300 K with Γ-point restricted sampling. The topological phase was then identified by calculating the Z_2_ topological invariant number with the Z2Pack Package.^59,60^ The tight-binding Hamiltonians were constructed using the Wannier90 ^61^ program, and the topological edge states were obtained using iterative Green’s function using the Wannier Tools package.^62^ Based on the constructed Wannier functions, we also calculated the evolution of Wannier Charge Centers (WCC) following the method for systems without inversion symmetry^59^ to verify the Z_2_ topological invariant number. The Wannier90 package was used together with the Quantum Espresso^63^ (QE) software to calculate the Berry curvature and spin Hall conductivity under the HSE06 functional. The plane-wave kinetic-energy cutoff and the charge density energy cutoff were set to 80 and 640 Ry, respectively. The convergence threshold for self-consistency was set to 10^–8^ Ry. A mesh grid of 21 × 21 was used to plot the spin texture using the PyProcar package.^64^

## Results and Discussion

### Structural Properties

In this work, we created a total of 22 structures (16 pristine structures and 6 Janus structures). To begin with, we constructed wurtzite, zincblende, DLHC, and AA structural configurations for the pristine InA (A = P, As, Sb, or Bi) case as shown in Figures 1a, 1b, 1c, and 1d. The 2D zincblende structure is a truncated bulk zincblende structure, while the wurtzite, DLHC, and AA configurations were stacked as bilayers with hexagonal symmetry. Upon relaxing these structures, we found that wurtzite and zincblende structures in the ultrathin limit tend to relax to a DLHC structure, while the AA configuration exhibits a higher ground-state energy in comparison to that of the DLHC structure. The findings are presented in Table S1 of the Supporting Information. This table shows that all materials exhibit an energetic preference for the DLHC configuration. Upon confirming that the DLHC configuration is the energetically preferred structure, we further tailored a Janus structure based on this configuration, having a space group *P*3*m*1 (no. 156), by substituting the top layer of A atoms with B atoms (In_2_AB, where A, B = P, As, Sb, or Bi, and A ≠ B). The optimized lattice parameters for the DLHC configuration for pristine and Janus structures are summarized in Tables S2 and 1, respectively. Additionally, Figures 1e and 1f illustrate the side and top views of the pristine DLHC structure, while Figure 1g and 1h illustrate the side and top views of the Janus DLHC structure. Figure 1i represents the first Brillouin zone (BZ) with labeled high-symmetry points.

**Figure 1:**
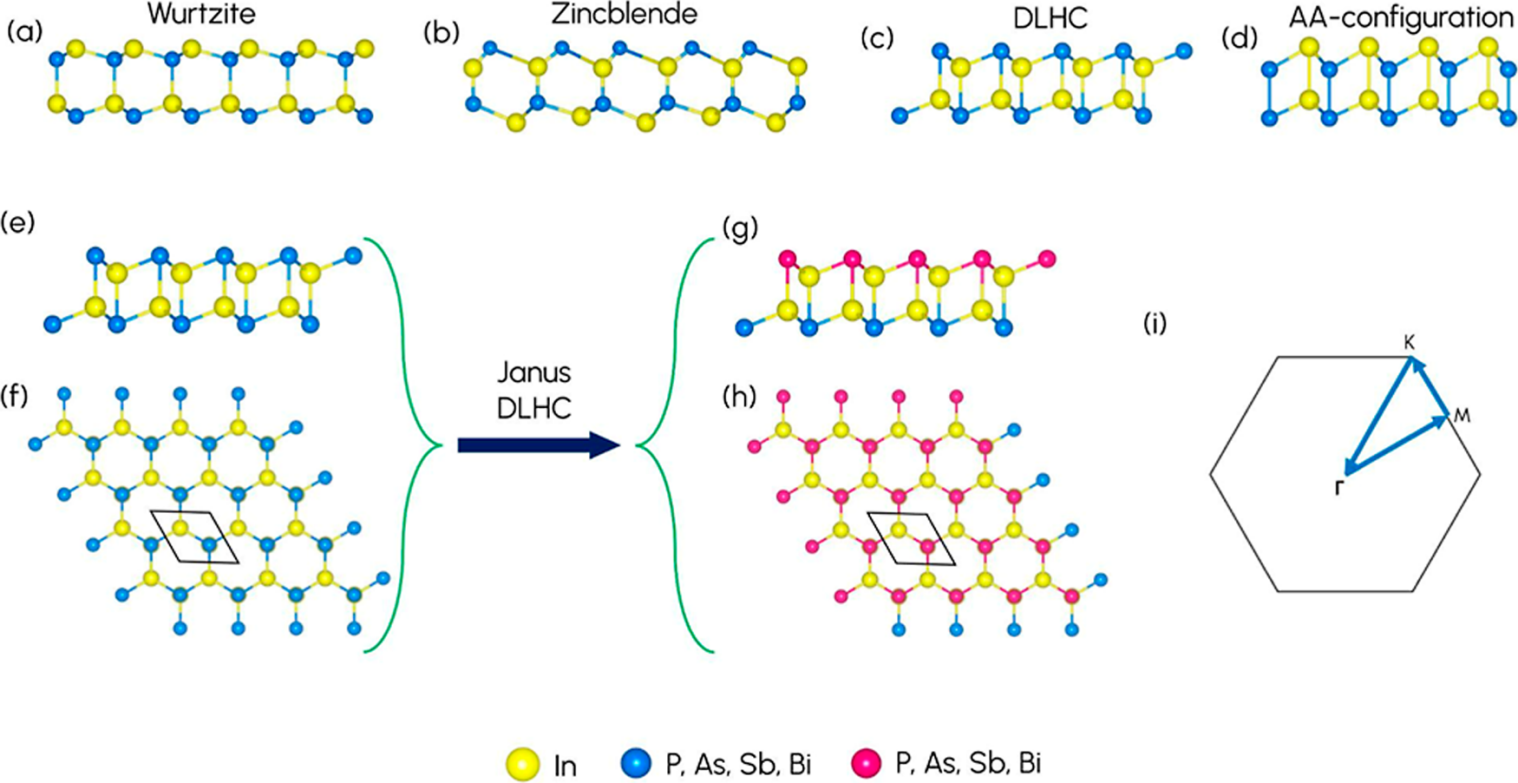
Crystal structures of (a) wurtzite, (b) zincblende, (c) DLHC, and (d) AA configurations. The side view (e) and the top view (f) of pristine DLHC. The side view (g) and the top view (h) of Janus DLHC. (i) High-symmetry points labeled first Brillouin zone (BZ) of the unit cell.

**Table 1:** Computed Lattice Parameters, System Bandgap, Bandgap at Γ, and Topological Z_2_ Invariant Number of Janus In_2_AB Structures Using GGA-PBE^a^

Material	Lattice Parameter, *a* = *b* (Å)	Bandgap (meV)	Bandgap at Γ (meV)	*Z*_2_ Invariance
In_2_PAs	4.32	65 (225)	191 (225)	1(0)
In_2_PBi	4.49	–47 (53)	1246 (1423)	1(0)
In_2_PSb	4.46	–41 (74)	439 (527)	1(1)
In_2_AsSb	4.49	21 (47)	400 (456)	1(1)
In_2_AsBi	4.55	–41 (37)	1148 (1298)	1(0)
In_2_BiSb	4.66	32 (117)	643 (649)	1(1)

a A negative system bandgap indicates that the valence band maximum is higher than the bottom of the conduction band minimum in energy. The values in the parentheses are calculated using HSE06. Topologically trivial phases are indicated by *Z*_2_ = 0, whereas nontrivial phases are *Z*_2_ = 1.

### Electronic and Topological Properties

After determining the energetically preferred structure, we first studied the electronic properties of pristine DLHC InA. The band structures for InP, InAs, InSb, and InBi using the GGA-PBE functional without and with SOC are shown in Supporting Information Figure S1. Their corresponding bandgaps are summarized in Table S2. Then, to determine the topological properties, we calculated their *Z*_2_ topological invariant number, where a material with *Z*_2_ = 1 signifies a nontrivial topological phase, whereas *Z*_2_ = 0 indicates a trivial material. However, our calculations showed that none of the compounds in their pristine state exhibit nontrivial topology. We then repeated the same procedure on the tailored inversion-symmetry-broken Janus In_2_AB structures (see Supporting Information Figure S2), and interestingly, under GGA-PBE, all six compounds exhibited nontrivial topological nature as summarized in Table 1. Given that GGA-PBE is known to underestimate the bandgaps, a hybrid functional approach (HSE06) was employed for more accurate bandgap predictions. The band structures obtained using HSE06 with the inclusion of SOC for all six Janus compounds are presented in Figure 2, and the HSE06 band structures without and with the inclusion of SOC are shown in Supporting Information Figures S3 and S4, while their corresponding system bandgaps and bandgaps at Γ are summarized in Table 1. After the inclusion of spin–orbit coupling (SOC) under HSE06, all six compounds In_2_PAs, In_2_PBi, In_2_PSb, In_2_AsSb, In_2_AsBi, and In_2_BiSb were found to be insulating with narrow bandgaps of 225 meV, 53 meV, 74 meV, 47 meV, 37 meV, and 117 meV, respectively. We also recalculated the Z_2_ invariant of all six Janus structures using HSE06. Notably, three compounds (In_2_PSb, In_2_AsSb, and In_2_BiSb) maintained their Z_2_ value of 1, confirming that they are topologically nontrivial, as summarized in Table 1.

**Figure 2:**
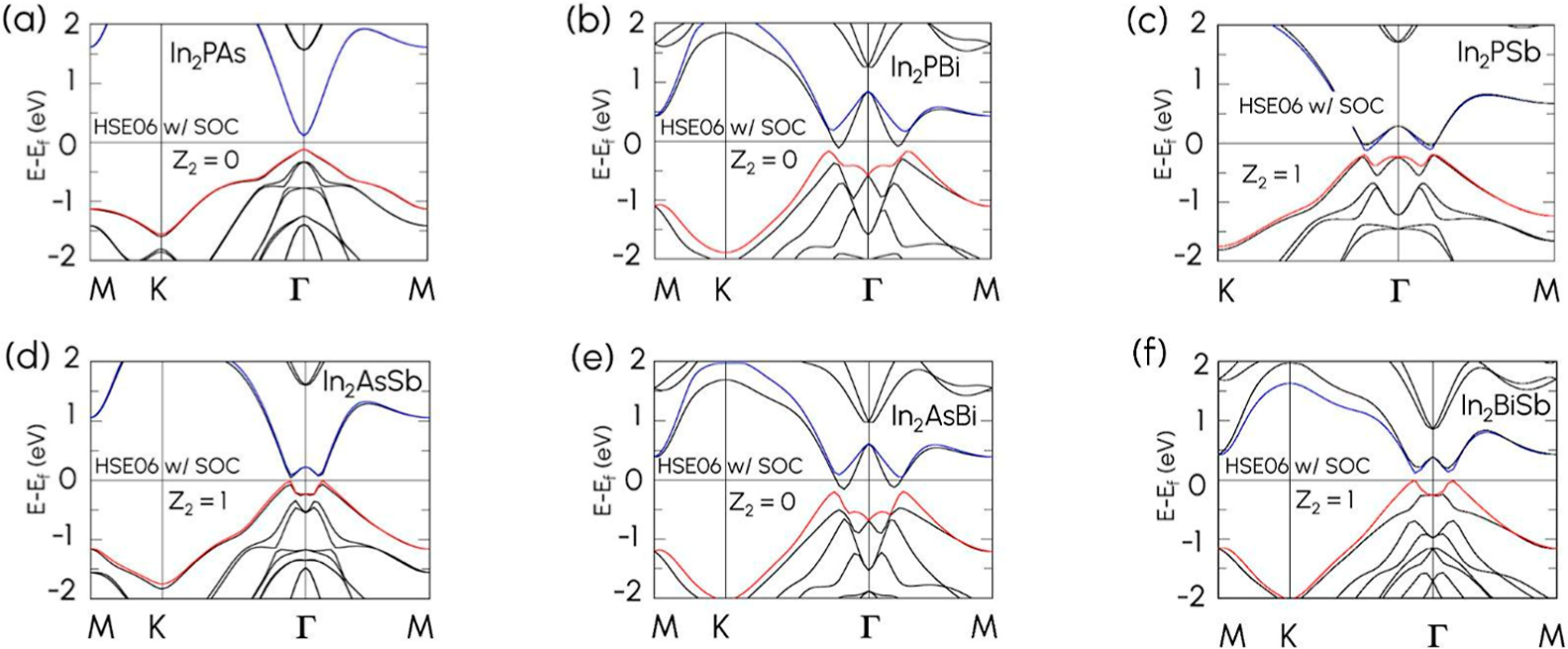
Band structures of (a) In_2_PAs, (b) In_2_PBi, (c) In_2_PSb, (d) In_2_AsSb, (e) In_2_AsBi, and (f) In_2_BiSb under HSE06 with SOC. The blue line corresponds to the conduction band minimum (CBM), while the red line corresponds to the valence band maximum.

To gain further insight into the topological properties, among the three materials that exhibit nontrivial topological properties (In_2_PSb, In_2_AsSb, and In_2_BiSb), we selected In_2_AsSb as the representative material. While all three materials display topologically protected edge states, the In_2_AsSb structure exhibits the clearest edge state in our calculations. Figures 3a and 3b show the electronic band structures of In_2_AsSb under HSE06 without and with the inclusion of SOC, respectively. We see in Figure 3a that without the inclusion of SOC under HSE06, the valence band maximum (VBM) and the conduction band minimum (CBM) touch at the Γ-point. Interestingly, by including SOC, there is an opening at the Γ-point, as seen in Figure 3b.

**Figure 3:**
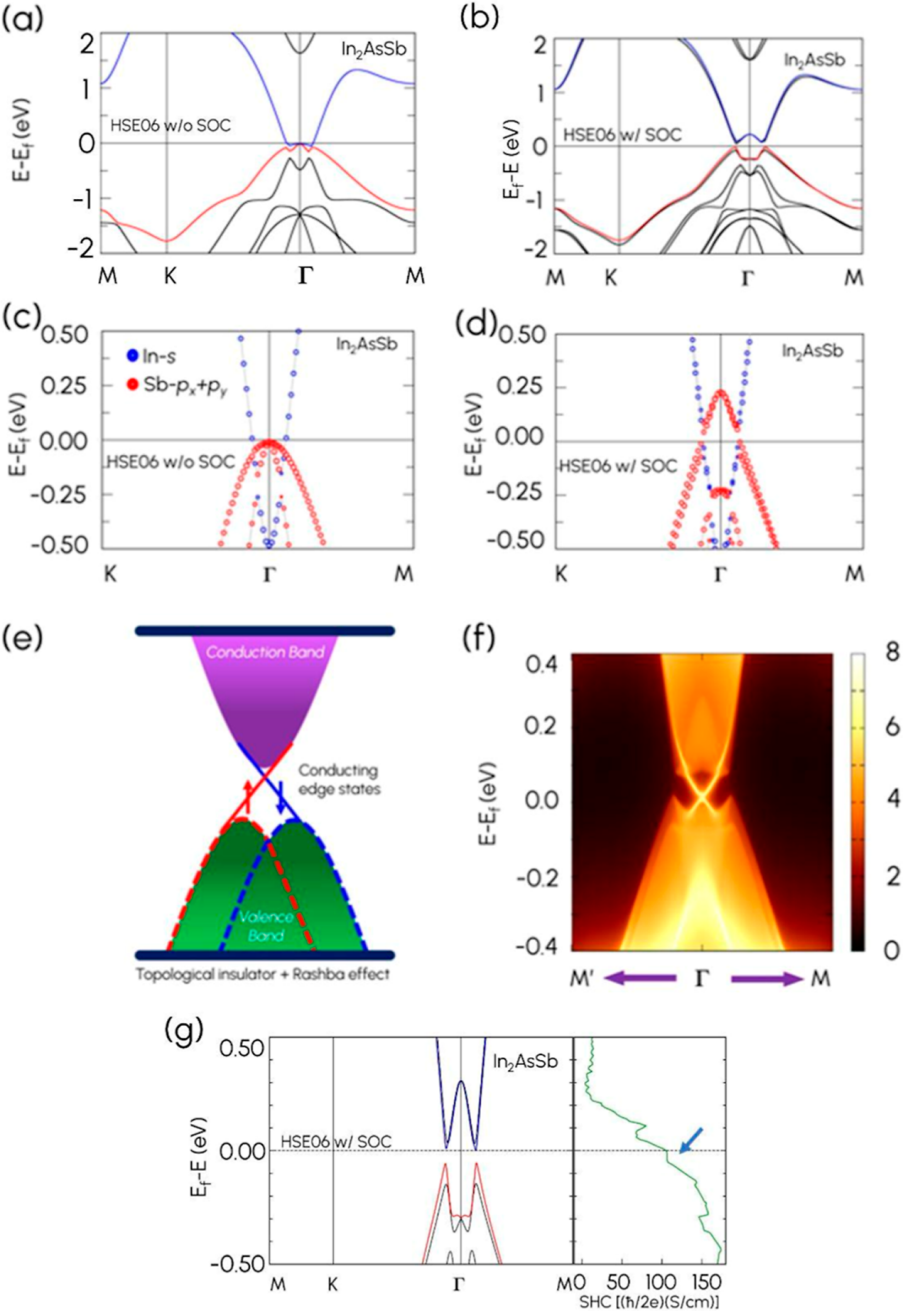
Band structures for In_2_AsSb using the HSE06 functional (a) without SOC and (b) with SOC. The red line corresponds to the valence band maximum (VBM), while the blue line represents the conduction band minimum (CBM). Band structures with orbital projections of In_2_AsSb under HSE06 (c) without and (d) with SOC, respectively. (e) Illustration of a gapless edge state. (f) Gapless edge state for In_2_AsSb. (g) The spin Hall conductivity (SHC) for In_2_AsSb.

To further investigate the mechanism underlying the nontrivial topological phase, we conducted a detailed orbital analysis of In_2_AsSb. The partial band projections, presented in Figures 3c and 3d, reveal that the dominant contributions near the Fermi level originate primarily from the In-s (blue) and Sb-p_*x*_ *+* p_*y*_ (red) orbitals. Moreover, a topologically nontrivial system should exhibit an edge state, as illustrated in Figure 3e. Hence, to further confirm the nontrivial phase, edge states were calculated for In_2_AsSb under HSE06. Notably, in Figure 3f, we observe a gapless edge state for In_2_AsSb, which spans the bulk energy gap and links the valence band to the conduction band. We calculated the spin Hall conductivity (SHC) to confirm the nontrivial topologies in In_2_AsSb further. As shown in Figure 3g, we observed the SHC plateaus precisely within the nontrivial bandgap region around the Fermi level, indicating that the SHC is quantized. The computed SHC has a quantized value of σ_*xy*_ ∼ 100 (h̵/2e)­(S/cm). This result provides direct evidence of the nontrivial topological nature of our system, confirming the conclusion from the Z_2_ invariant and edge states. Additionally, the calculation of Wannier charge centers (WCC) in Supporting Information Figure S7 shows that the number of crossings on the largest gaps between the WCC and any reference straight line is odd, confirming the conclusion from the *Z*_2_ invariant and edge states. This proves that In_2_AsSb has a nontrivial topological phase, and the gapless edge state for In_2_PSb and In_2_BiSb could be seen in Supporting Information Figure S6.

Beyond the topological insulating phase in Janus In_2_AB structures, interestingly, we also observed the presence of Rashba-like spin splitting in the Janus In_2_PSb DLHC structure, which is a direct consequence of symmetry breaking.

As seen in the band structure, since In_2_PSb is the only material exhibiting observable spin-splitting under PBE + SOC and HSE06 + SOC, we will be using it as the representative material for further analysis of the Rashba effect. Figure 4a shows spin-up and spin-down chiral states, while Figure 4b gives a schematic description of Rashba splitting. Figure 4c shows the band structure calculated by using GGA-PBE with SOC. A detailed analysis reveals that In_2_PSb exhibits Rashba splitting at the VBM near the Γ-point upon incorporation of SOC. Also, from Figure 4c, it is clear that SOC induces band splitting along the high-symmetry path K–Γ–M.

**Figure 4:**
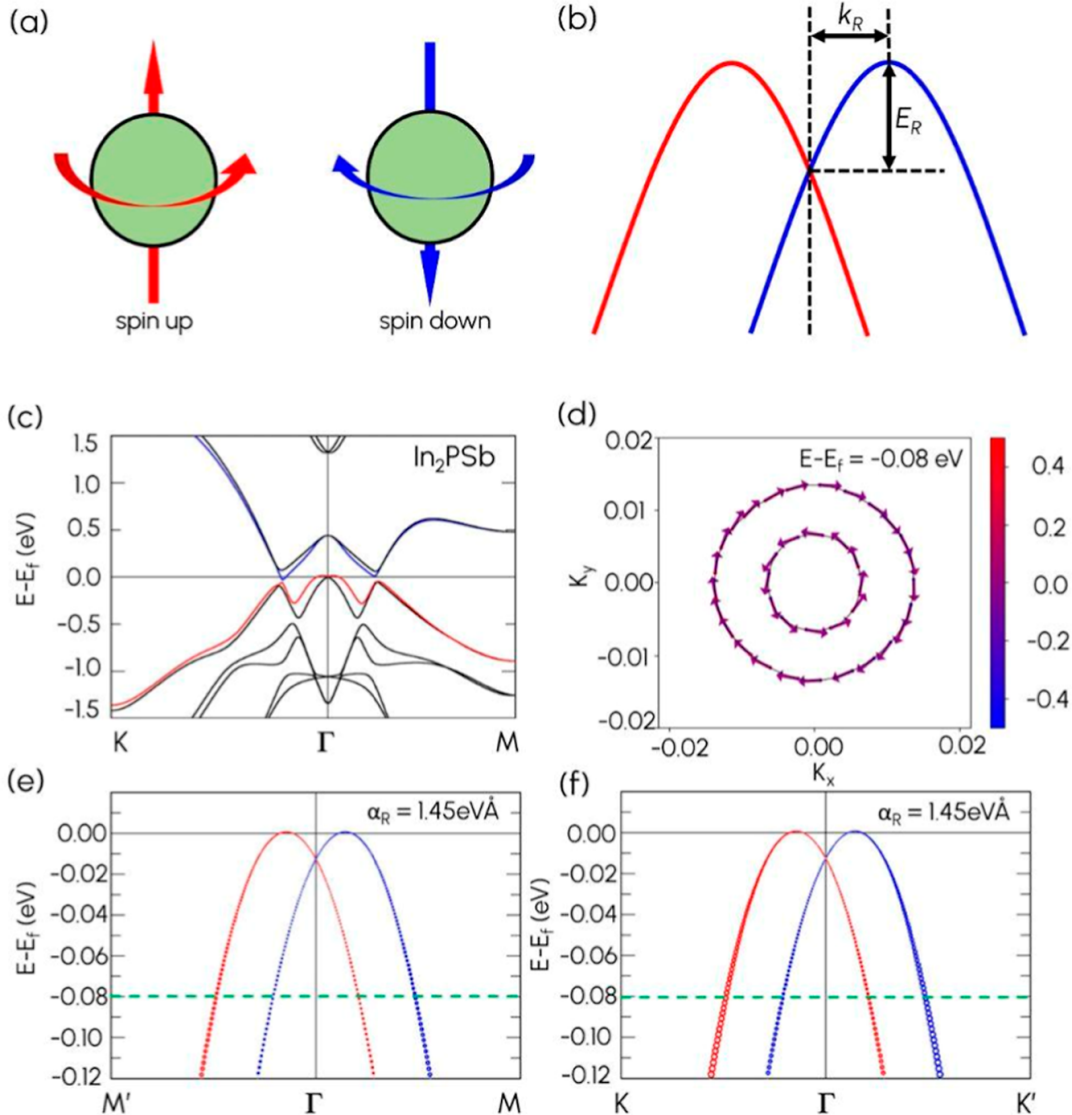
Rashba splitting in 2D Janus In_2_PSb. (a) Spin-up and spin-down chiral states. (b) A schematic description of Rashba splitting with the momentum offset *k*_R_ and energy *E*_R_. (c) PBE + SOC band structure. (d) 2D spin texture of In_2_PSb sliced at *E* – *E*_f_ = −0.08 eV. The spin-projected bands centered at Γ along (e) M′–Γ–M with red and blue circles corresponding to S_*x*_^+^ and S_*x*_^–^, respectively, and along (f) K–Γ–K′ with red and blue circles corresponding to S_*y*_^+^ and S_*y*_^–^, respectively.

### Spin Properties

To gain a deeper insight into the Rashba bands’ spin configuration, we generated a 2D spin texture at an energy slice of *E* – *E*_f_ = −0.08 eV. The green dotted line in Figures 4e and 4f marks the energy level corresponding to this slicing. In Figure 4d, the arrows represent the in-plane vector components S_*x*_ and S_*y*_; the presence of clockwise and counterclockwise spin rotations depicts the distinctive feature of the Rashba effect. In comparison, the red-blue gradient depicts the out-of-plane S_*z*_ vector component. Furthermore, the spin propagation is centered at the Γ-point below the Fermi level *E*_f_. Figure 4d shows the spin texture at the Γ-point, where the inner and outer bands exhibit counter-rotating patterns. This opposite alignment of the in-plane spin components (S_*x*_, S_*y*_) is a defining feature of the Rashba effect. Also, we calculated the strength of the Rashba spin-splitting using the formula α_R_ = 2*E*_R_/*k*_R_. The resulting Rashba parameters along K–Γ–K′ (α_R_^K−Γ^) and along M′–Γ–M (α_R_^Γ–M^) are both 1.45 eV Å, indicating a significant isotropic Rashba splitting.

While Janus Al_2_SbBi has the highest reported Rashba parameter of 3.31 eV Å^45^ among the DLHCs, our results show a substantial Rashba parameter of 1.45 eV Å for In_2_PSb, making it a promising candidate in its own right.

### Thermodynamic Stability

The dynamic stability was assessed through phonon dispersion calculations. The resulting phonon spectra are presented in Figure 5 and Supporting Information Figure S8. Notably, there are no significant imaginary frequencies for In_2_AsSb and In_2_PSb structures, as seen in Figures 5b and 5d, confirming the dynamic stability of these structures. Along with the dynamic stabilities, we calculated the thermal stabilities of both representative structures using ab initio molecular dynamics (AIMD) simulations. From the AIMD simulations at 300 K, we found that both In_2_AsSb and In_2_PSb do not exhibit any structural reconstruction, indicating that both In_2_AsSb and In_2_PSb are thermally stable. The total energy fluctuation as a function of time for In_2_AsSb and In_2_PSb is given in Figures 5a and 5c, respectively. Thus, the phonon dispersion and AIMD results show that In_2_AsSb and In_2_PSb structures are thermodynamically stable, which indicates the possibility of experimental synthesis.

**Figure 5:**
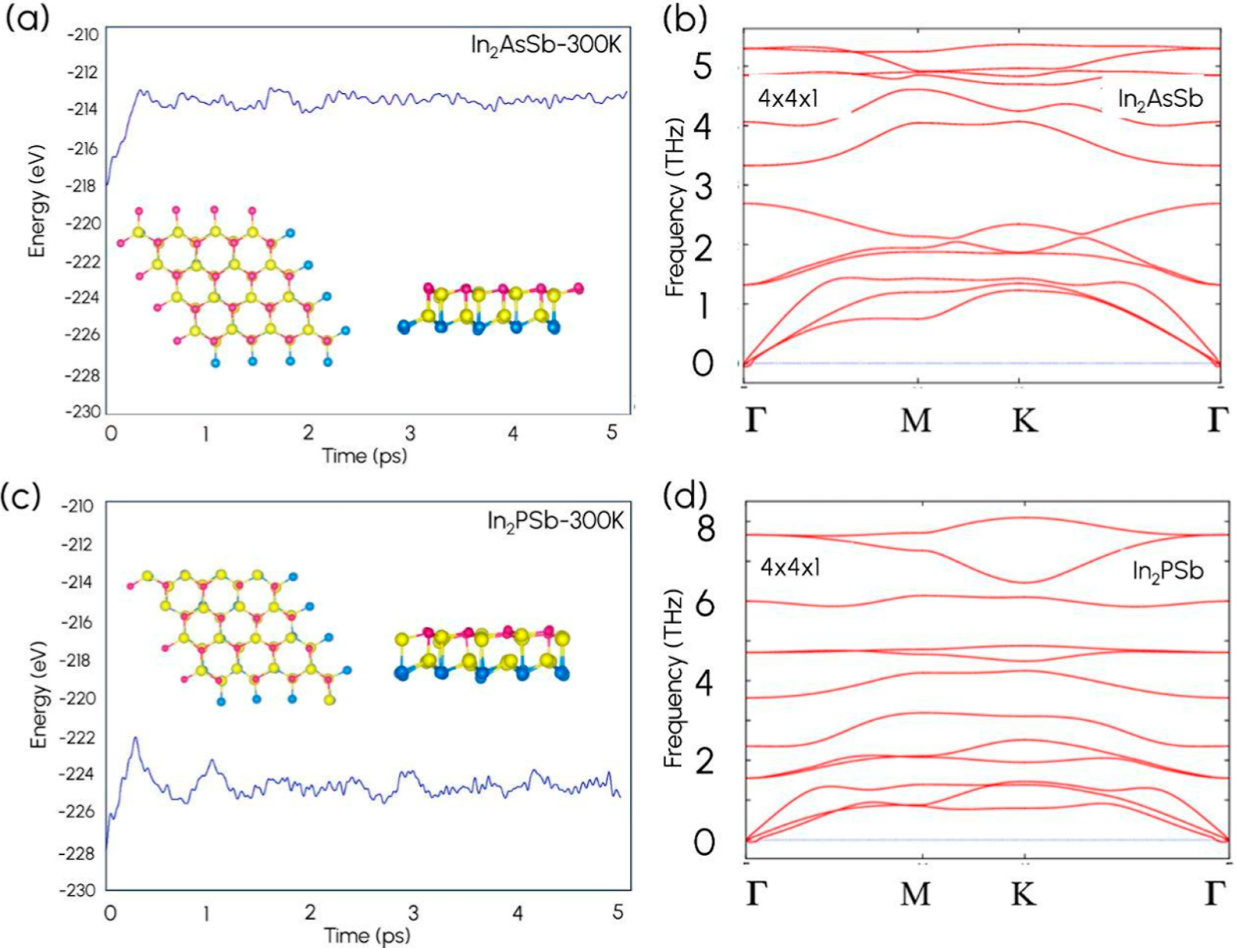
Total energy fluctuation with time using the AIMD simulation of (a) In_2_AsSb and (c) In_2_PSb at 300 K. The top view and the side view of the structure during the simulation are indicated as the inset. Phonon dispersion spectra of (b) In_2_AsSb and (d) In_2_PSb structures.

## Conclusion

In conclusion, we demonstrated that pristine InA structures prefer DLHC structures compared to wurtzite, zincblende, and bilayer configurations. Additionally, with this energetically preferred configuration, we constructed Janus In_2_AB structures. Using first-principles calculations, we investigated the structural stability and electronic and topological properties of six In_2_AB structures. Remarkably, three structures, namely, In_2_PSb, In_2_AsSb, and In_2_BiSb, were found to have nontrivial topological phases, with bandgaps of 527, 456, and 649 meV, respectively. For In_2_AsSb, contributions primarily from the In-s and Sb-p_*x*_ + p_*y*_ orbitals were dominant. The nontrivial topological properties were confirmed by the presence of gapless edge states. Along with the nontrivial topological properties, In_2_PSb exhibited strong isotropic Rashba splitting (α_R_^K−Γ^ = α_R_^Γ–M^ = 1.45 eV Å). AIMD simulations at 300 K and phonon dispersion confirmed the thermodynamic stabilities of In_2_AsSb and In_2_PSb. These findings show that the materials that break inversion symmetry exhibit a nontrivial topological phase, and along with this, some materials also exhibit Rashba-type splitting in In_2_AB structures, demonstrating their potential for future spintronic applications.

## Supplementary Material



## Notes

Ground-state energies, phonon dispersion spectra, band structures with GGA-PBE and HSE06, and edge states (PDF)
