# Supplementary material for: In2AB Janus Double-Layered Honeycomb Structures with Nontrivial Topology and Rashba Splitting (A, B = P, As, Sb, or Bi)
Source: ACS Omega. 2025 Nov 10;10(46):56217–24. doi: 10.1021/acsomega.5c07907 (PMC12658597; doi:10.1021/acsomega.5c07907)
Supplement: Supplementary file 1 [file ao5c07907_si_001.pdf]

Supporting Information for

In_2AB Janus Double Layered HoneyComb Structures with Nontrivial Topology and Rashba Splitting (A, B = P, As, Sb, or Bi)

Joel D'Souza[1], Ina Marie R. Verzola[1], Rovi Angelo B. Villaos[1,2,3], Aniceto B. Maghirang III[1,4], Zhi-Quan Huang[1], Yijun Shi[3] and, Feng-Chuan Chuang[1,4,5,6*]

[1] *Department of Physics, National Sun Yat-sen University, Kaohsiung, 80424 Taiwan*

[2] *Division of Machine Elements, Luleå University of Technology, Luleå SE-97187, Sweden*

[3] *Applied Physics, Division of Materials Science, Department of Engineering Sciences and Mathematics, Luleå University of Technology, Luleå SE-97187, Sweden*

[4] *Physics Division, National Center for Theoretical Sciences, Taipei, 10617 Taiwan*

[5] *Center for Theoretical and Computational Physics, National Sun Yat-sen University, Kaohsiung, 80424 Taiwan*

[6] *Department of Physics, National Tsing Hua University, Hsinchu, 30013 Taiwan*

*Corresponding Author: Feng-Chuan Chuang

Postal Address: 70 Lienhai Rd., Kaohsiung 80424, Taiwan.

Telephone: +886-7-5253733

E-mail Address: fchuang@mail.nsysu.edu.tw

Table S1. Ground state energies of the pristine structures in Wurtzite, Zincblende, DLHC, and Bi-layer configurations.

Material	Wurtzite (eV)	Zincblende (eV)	DLHC (eV)	AA-configuration (eV)
InP	Relaxes to DLHC	Relaxes to DLHC	-15.9202	-14.6938
InAs	Relaxes to DLHC	Relaxes to DLHC	-14.6870	-13.7079
InSb	Relaxes to DLHC	Relaxes to DLHC	-13.4111	-12.6970
InBi	Relaxes to DLHC	Relaxes to DLHC	-14.2015	-13.6223

Table S2. Calculated lattice parameters, system band gap, band gap at Γ, and topological Z_2 invariant number of energetically preferred pristine monolayers using GGA-PBE. The trivial phases are indicated by $Z_2 = 0$ while the non-trivial phases are $Z_2 = 1$

Material	Energetically Preferred Structure	Lattice Parameter, a=b (Å)	Band Gap (meV)	Band Gap at Gamma (meV)	Z_2 PBE
InP	DLHC	4.26	168	168	0
InAs	DLHC	4.36	34	173	0
InSb	DLHC	4.62	125	318	0
InBi	DLHC	4.70	111	334	0

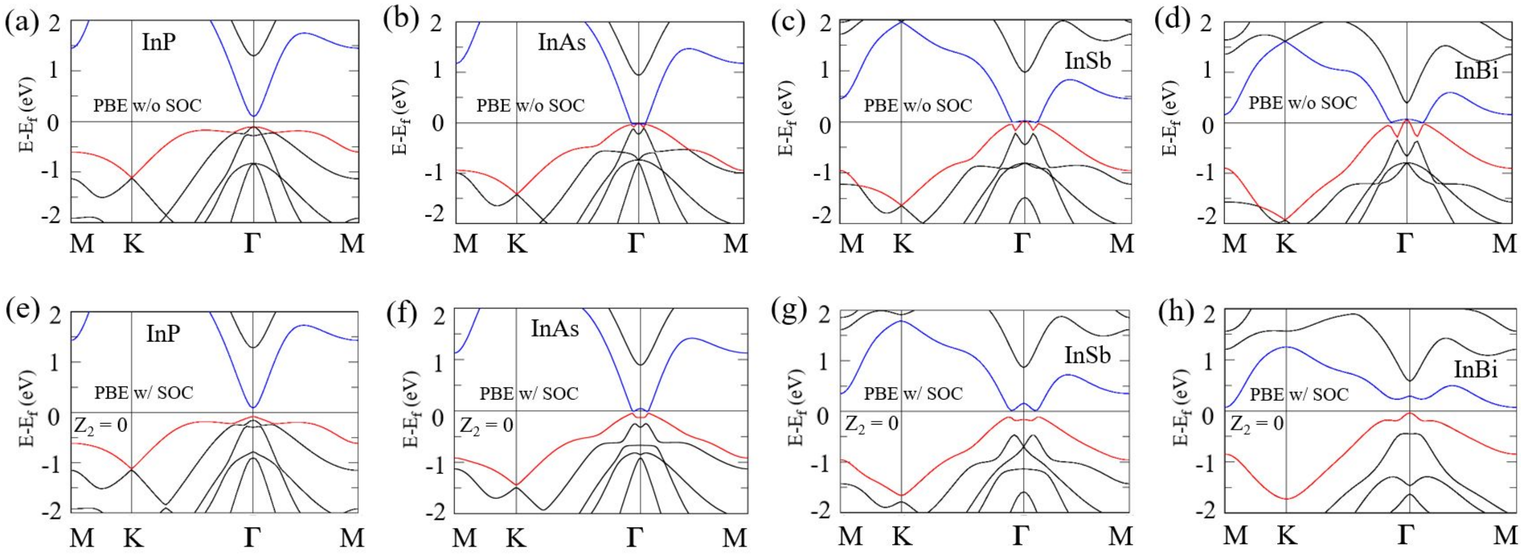

Figure S1. Band structures of InP, InAs, InSb, and InBi under GGA-PBE (a), (b), (c), and (d) without SOC and (e), (f), (g), and (h) with SOC. The blue line corresponds to the conduction band minimum (CBM), while the red line corresponds to the valence band maximum.

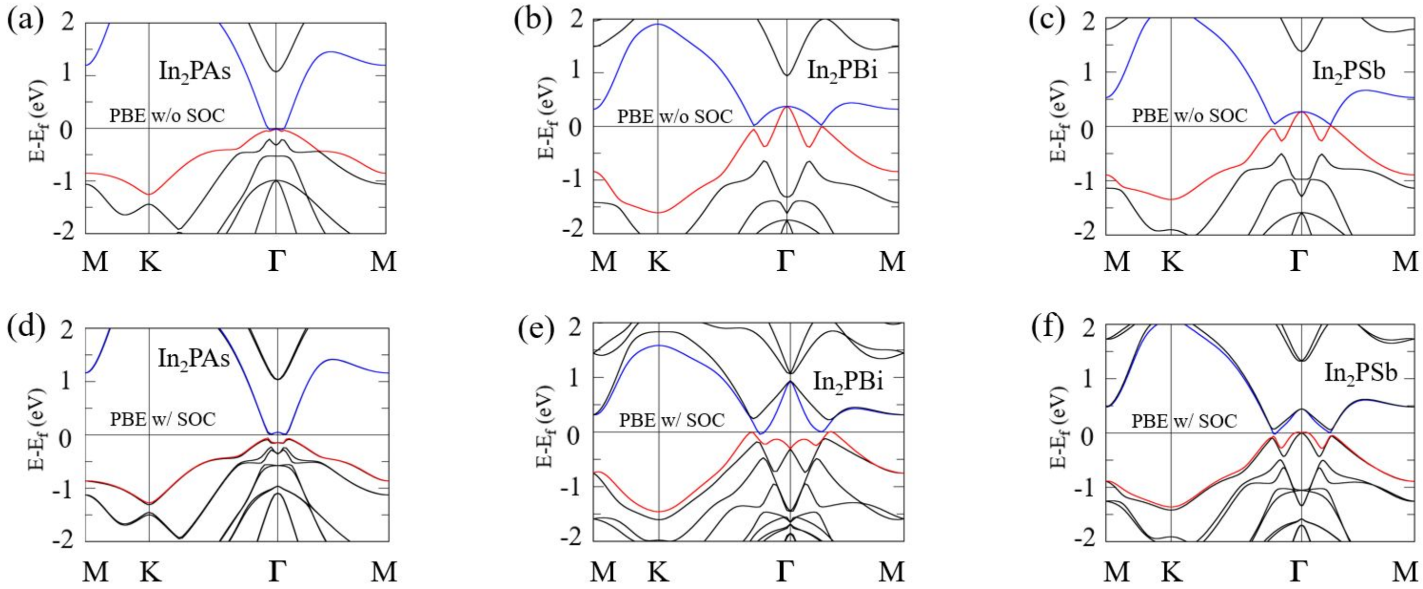

Figure S2. Band structures of In_2PAs, In_2PBi, and In_2PSb under GGA-PBE (a), (b), and (c) without SOC and (d), (e), and (f) with SOC

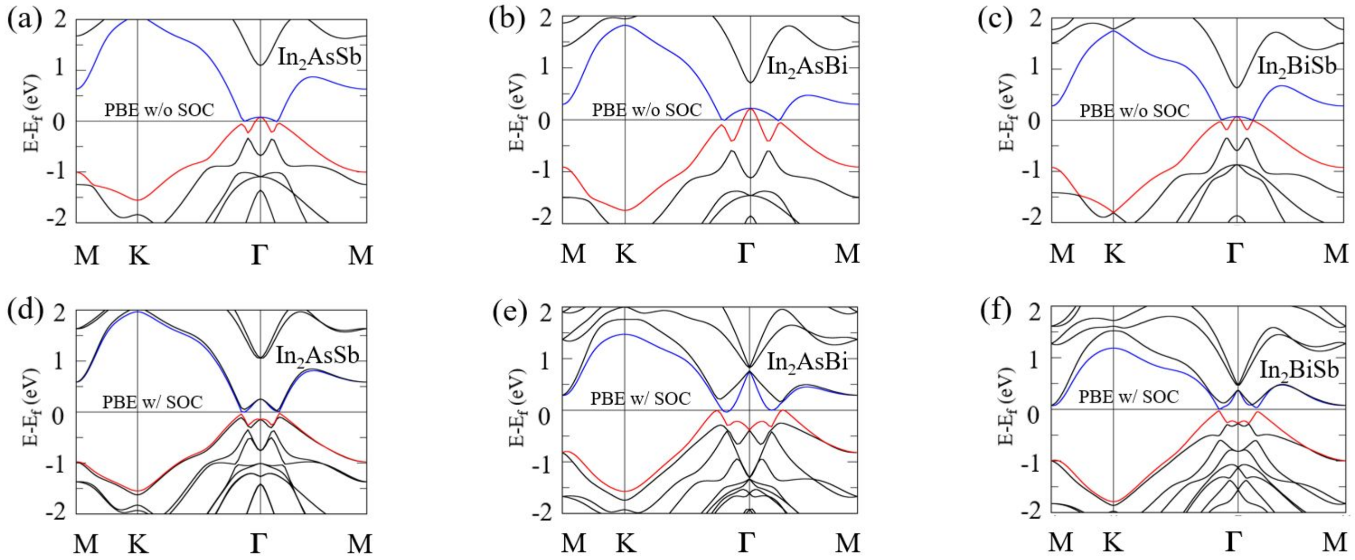

Figure S3. Band structures of In_2AsSb, In_2AsBi, and In_2BiSb under GGA-PBE (a), (b), and (c) without SOC and (d), (e), and (f) with SOC

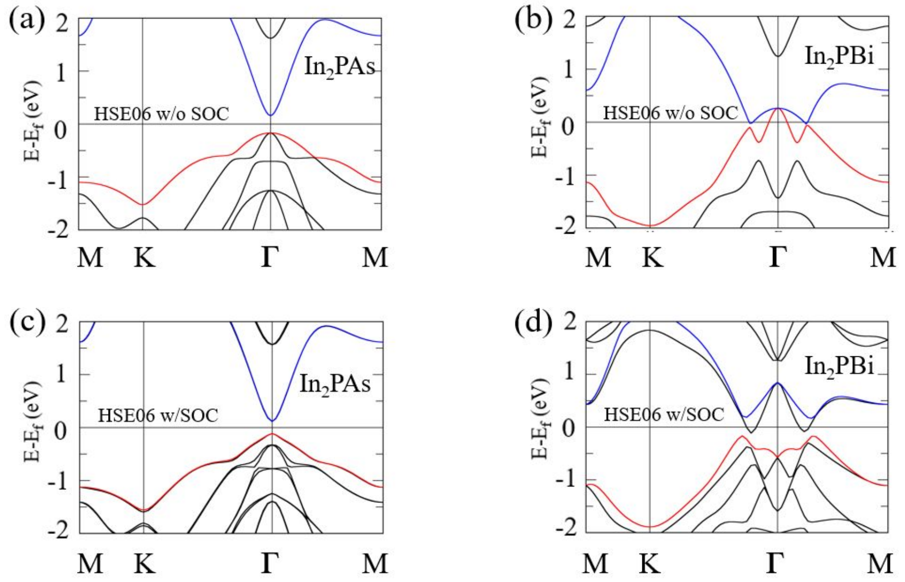

Figure S4. Band structures of In_2PAs, and In_2PBi under HSE06 (a), (b) without SOC and (c), (d) with SOC

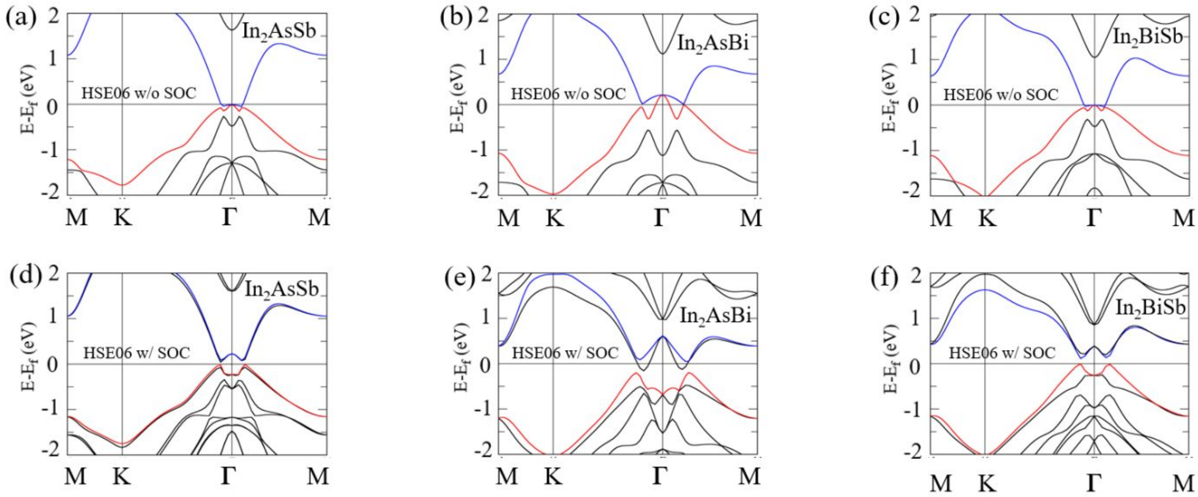

Figure S5. Band structures of In_2AsSb, In_2AsBi, and In_2BiSb under HSE06 (a), (b), and (c) without SOC and (d), (e), and (f) with SOC

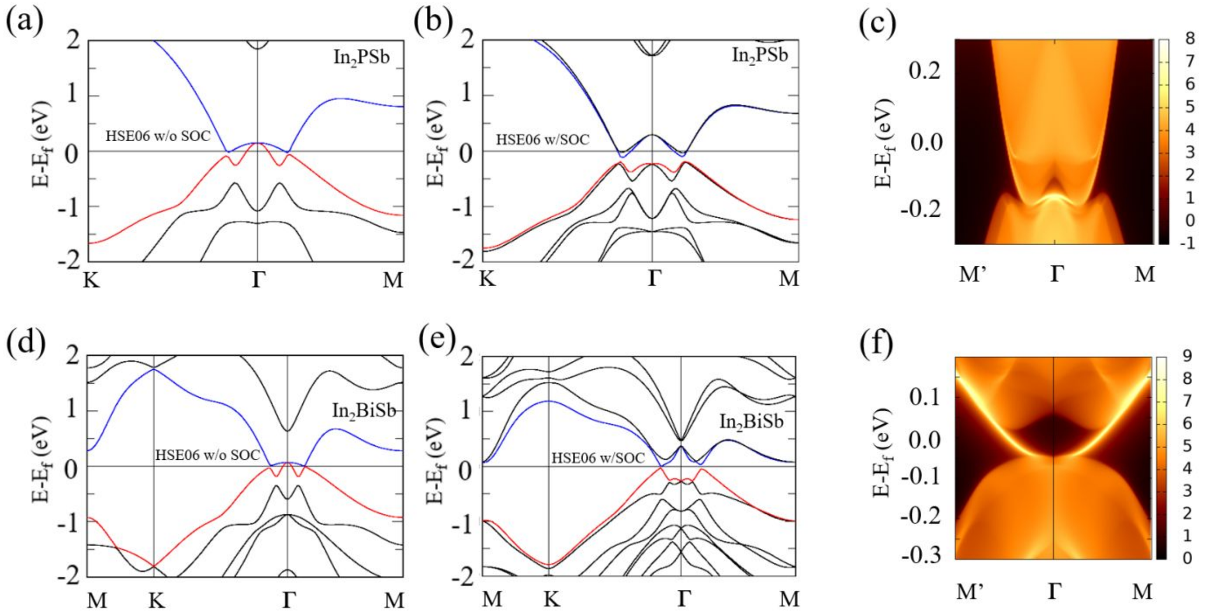

Figure S6. The band structures for In_2PSb and In_2BiSb using the PBE and HSE06 functional (a), (d) without SOC and (b), (e) with SOC. The red line corresponds to the valence band maximum (VBM) while the blue line represents the conduction band minimum (CBM). (c),(f) are the gapless edge states for In_2PSb and In_2BiSb.

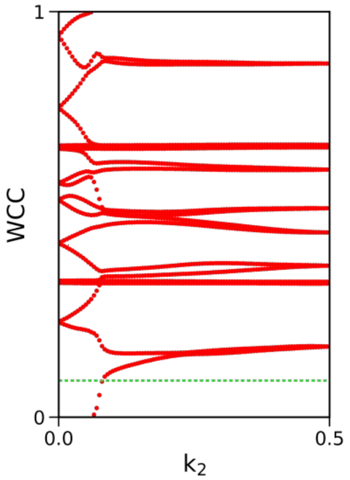

Figure S7. Evolution of the Wannier Charge Centers (WCC) along k_y (k_2) for In_2AsSb.

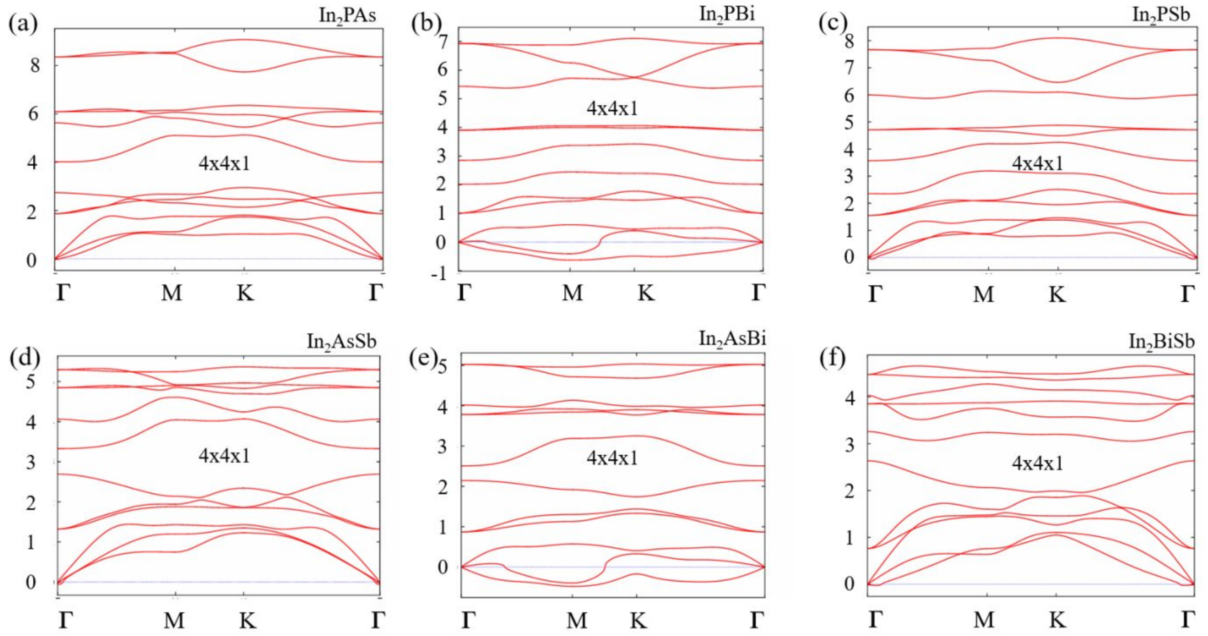

Figure S8. Phonon dispersion spectra of (a)In_2PAs, (b) In_2PBi, (c) In_2PSb, (d) In_2AsSb, (e) In_2AsBi, and (f) In_2BiSb monolayers with a 4x4x1 supercell.
